# Impact of a reduced dose intensity of adjuvant anthracycline based chemotherapy in a population-based cohort of stage I–II breast cancers

**DOI:** 10.3332/eCMS.2008.63

**Published:** 2008-07-08

**Authors:** AV Tinker, C Speers, J Barnett, IA Olivotto, S Chia

**Affiliations:** 1 Department of Medical Oncology, British Columbia Cancer Agency, Vancouver, BC, Canada; 2 Breast Cancer Outcomes Unit, British Columbia Cancer Agency, Victoria, BC, Canada; 3 Department of Pharmacy, British Columbia Cancer Agency, Victoria, BC, Canada; 4 Department of Radiation Oncology, British Columbia Cancer Agency, Victoria, BC, Canada

## Abstract

**Background:**

Reductions in the dose intensity (DI) of adjuvant anthracycline-based chemotherapy in early stage breast cancer are frequently required due to treatment toxicity or poor tolerance, but the implications of a minimal reduction in DI on clinical outcome remain uncertain.

**Patients and methods:**

Women with stage I–II breast cancer treated with adjuvant adriamycin and cyclophosphamide (AC) from 1990–95 were identified in a provincial breast cancer database. Cases were classified into four cohorts: (1) all cycles delivered at full dose and on time; (2) one single dose reduction or dose delay; (3) >1 dose reduction or dose delay; (4) <2 cycles of chemotherapy delivered.

**Results:**

484 eligible cases were identified (cohort (1): *n* = 268; (2): *n*= 88; (3): *n*= 89; (4) *n*= 39). Slight imbalances in lymph node status (p=0.05) and adjuvant hormonal therapy (p=0.05) were observed between the cohorts. Fifty-five per cent (267/484) of the patients had node-positive disease and 33% (158/484) were ER+. 45% of cases had a reduction in DI. With a median follow-up of 9.6 years, there were no significant differences in relapse-free survival (p=0.94), breast cancer-specific survival (p=0.87) or overall survival (p=0.86) between the four cohorts. Outcomes were independent of hormone receptor status.

**Conclusions:**

Although toxicity related reductions in the DI of adjuvant AC chemotherapy for early stage breast cancer are common, they did not appear to significantly impact on clinical outcomes in this population-based cohort of women with stage I–II breast cancers.

## Introduction

The benefits of adjuvant chemotherapy on clinical outcomes in early stage breast cancer have been well established [1, 2]. In the latest Early Breast Cancer Trialists’ Collaborative Group overview of the randomized trials in early breast cancer, anthracyclines were shown to be superior to non-anthracyclines in reducing recurrences and breast cancer deaths [1]. The concept of dose intensity (DI), defined as the amount of drug delivered per unit time (mg/m^2^/week), and its impact on breast cancer outcomes has been an area of intensive research. The French Adjuvant Study Group (FASC) 05 trial compared high and low (50%) DI of epirubicin in a combination containing fluorouracil, epirubicin and cyclophosphamide [3]. The higher DI arm yielded significant improvements in disease free survival (DFS) and overall survival (OS). Similarly, the Cancer and Leukaemia Group B (CALGB) 8541 trial compared high, intermediate and low DI of doxorubicin, using the combination of cyclophosphamide, doxorubicin and fluorouracil [4]. Women treated at the high and intermediate DI had significantly improved DFS and OS over the low DI group. An ensuing CALGB study (9344) found that anthracycline DI exceeding the highest used in the CALGB 8541 study (60 mg/m^2^ every 21 days) failed to further improve survival but was associated with increased treatment-related toxicity [5]. Furthermore, a dose effect of escalating cyclophosphamide has not been identified as beneficial in two randomized trials [6, 7]. Taken together these trials demonstrate that within the standard anthracycline dose range a threshold effect exists, meaning it is less efficacious to deliver adjuvant chemotherapy, using sub-optimal dose intensity and/or lower cumulative doses of anthracyclines. In order to maximally improve survival for women with early stage breast cancer, a critical (or threshold) dose intensity and/or cumulative anthracycline dose must be reached.

It remains controversial at what threshold a reduction in DI, other than ≤50%, adversely impacts on clinical outcomes. A retrospective analysis of the pivotal Milan trial using classical cyclophosphamide methotrexate fluorouracil (CMF) suggests that the women who received less than 85% of their scheduled dose had worse clinical outcomes [8, 9]. In addition, women who received less than 65% of their scheduled dose did no better than those treated with surgery alone. However, more recent retrospective data from larger cohorts of women treated with classical or intravenous CMF have failed to demonstrate a statistically significant correlation between DI of chemotherapy and clinical outcomes [10, 11].

Reduced dose intensity of adjuvant chemotherapy because of toxicity or poor treatment tolerance in primary breast cancer is a common occurrence [12]. In a US-wide study of community practices with close to 20,000 women with early stage breast cancer treated with adjuvant chemotherapy, 55.5% of patients received a DI of less than 85%. Doxorubicin and cyclophosphamide (AC) is a commonly used combination for the treatment of early stage breast cancer. We have examined the impact of a reduced DI of four cycles of AC on clinical outcomes of patients with early stage breast cancer treated at the British Columbia Cancer Agency (BCCA) between 1990 and 1995.

## Methods

The province of British Columbia (BC) has a population of approximately four million people. In 2005, close to 2600 new cases of breast cancer were diagnosed, and 620 breast cancer related deaths were recorded. Approximately 75% of all cases of breast cancer diagnosed in the province are referred to a BCCA centre. The BCCA has the mandate for cancer control for the entire province. This includes the operation of four regional cancer centres delivering all the radiation therapy in the province, and the management of the provincial budget for all cancer systemic therapies. The BCCA is also responsible for the establishment of provincial, evidence-based guidelines regarding access to systemic agents in the treatment of all solid and haematological malignancies. A central pharmacy database within the BCCA records the date, drug and dose of all systemic agents and their indication in the treatment of a specific cancer in every patient. The BCCA also manages a Breast Cancer Outcomes Database (BCOD). The BCOD contains detailed demographic, pathologic, staging, treatment and outcome data for women diagnosed with breast cancer since 1 January 1989 and referred to the BCCA. Information on the date and site of first local, regional and distant relapse is collected prospectively. Date and cause of death is collected from the provincial death registry.

The BCCA pharmacy database and the BCOD were used to identify women with stage I–II breast cancer who were treated with standard adjuvant AC chemotherapy (A: 60 mg/m^2^; C: 600mg/m^2^; administered every 21 days for a total of four cycles) between 1990 and 1995. During this time period G-CSF was not routinely used for patients receiving adjuvant chemotherapy at the BCCA. Women with bilateral invasive breast cancers diagnosed within ten years of each other and more than one month apart were excluded from the study. The reasons for dose adjustments were not determined for this study, but are assumed to be related to treatment toxicity, patient preference and/or physician preference. The treatment data were then linked to the BCOD to determine clinical outcomes.

Standard BCCA guidelines for the management of treatment-related side effects state that with the AC regimen, both adriamycin and cyclophosphamide should each be reduced by 25% for mild myelosuppresion on day 1 of any treatment cycle (defined as: absolute neutrophil count (ANC) 1.0–1.49 × 10^9^/l and/or platelets 70–89 × 10^9^/l) or that treatment be delayed for more severe myelosuppresion on day 1 of any treatment cycle (defined as ANC < 1.0 × 10^9^/l and/or platelets <70 × 10^9^/l). Reductions in AC dose are recommended for either hyperbilirubinemia (adriamycin is reduced 50% if serum bilirubin is 20–51 μmol/l, 75% if the serum bilirubin is 51–85 μmol/l and discontinued if the serum bilirubin is >85 μmol/l) or severe renal dysfunction (cyclophosphamide is reduced by 25% if the creatinine clearance is <10 ml/min).

Reduced DI was defined as: (1) a 25% reduction from the starting dose of at least one of the chemotherapeutic agents (per cycle), or (2) a dose delay of at least five days (per cycle). The patients were then grouped into one of four cohorts: (1) entire treatment course delivered at full doses and on schedule; (2) one single dose reduction or one single dose delay during the entire treatment course; (3) >1 dose reduction or dose delay during the entire treatment course; and (4) ≤2 cycles of treatment delivered. The rationale in segregating dose intensity of AC into these four cohorts was based on our primary interest in assessing the outcome between cohorts 1 and 2. It appears clinicians often initiate growth factor support upon the first occurrence of either a dose reduction or delay of adjuvant chemotherapy without much clinical evidence to support this practice. The BCOD, provincial pharmacy database and uniform treatment guidelines allowed us to attempt to address this dilemma in a retrospective manner.

Relapse free survival (RFS) was defined as the time from initial pathologic diagnosis of the primary breast cancer until the first relapse (local, regional or distant) or death from breast cancer in the absence of a documented relapse. Overall survival (OS) was defined as the time from initial pathologic diagnosis of the primary breast cancer until death from any cause. Breast cancer specific survival (BCSS) was defined as the time from initial pathologic diagnosis of the primary breast cancer until death related to breast cancer. Where cause of death was unknown, it was attributed to breast cancer if there was a documented regional or distant relapse, or subsequent new primary contralateral breast cancer.

The χ^2^ statistic was used to compare categorical variables. Continuous variables were compared using analysis of variance. RFS, BCSS and OS rates were computed using the Kaplan-Meier method. Statistical comparisons of Kaplan-Meier curves were conducted using the log-rank test. Ethics approval was granted for this study from the BCCA research and ethics board.

No external funding was required for this study.

## Results

### Patient characteristics

Four-hundred-and-eighty-four cases were identified. Two-hundred-and-sixty-eight patients (55.4%) received their full course of treatment on time and at 100% DI (cohort 1). Eighty-eight patients (18.2%) had one dose reduction or dose delay (cohort 2) and 89 patients (18.4%) had more than one dose delay or dose reduction throughout treatment of at least three cycles of chemotherapy (cohort 3). Only 39 patients (8.1%) received two or fewer cycles of therapy (cohort 4).

The cohorts were well matched for characteristics of prognostic significance such as age, menopausal status, size of the primary lesion, tumour grade, oestrogen receptor status and surgical margins (Table 1). Nodal status (p=0.05) and use of adjuvant hormonal therapy (p=0.05) differed significantly between the cohorts. Sixty-six per cent of patients in cohort 2 had axillary node involvement, while only 56.2%, 47.7% and 46.2% had axillary node involvement in cohorts 1, 3 and 4, respectively. In cohort 2, 62.5% of women were prescribed adjuvant hormonal (e.g. tamoxifen) therapy, while only 48.9%, 42.7% and 46.2% women in cohorts 1, 3 and 4, respectively, were prescribed adjuvant hormonal therapy. The level of Her-2/***neu*** expression was not tested in patients diagnosed with primary breast cancer in the period of this study.

### Survival

**Figure 1:**
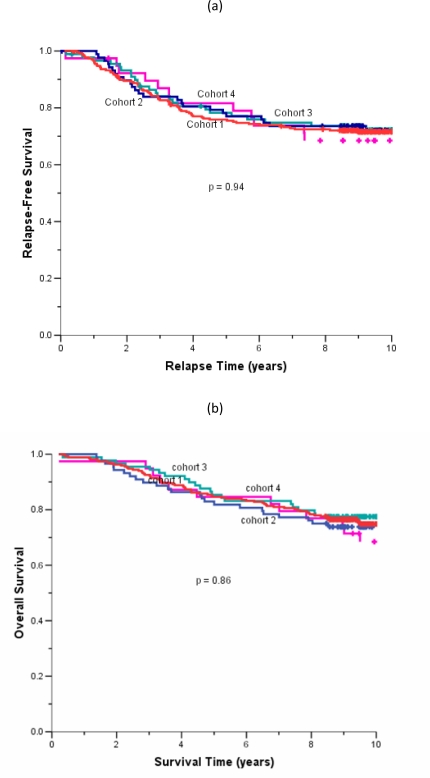

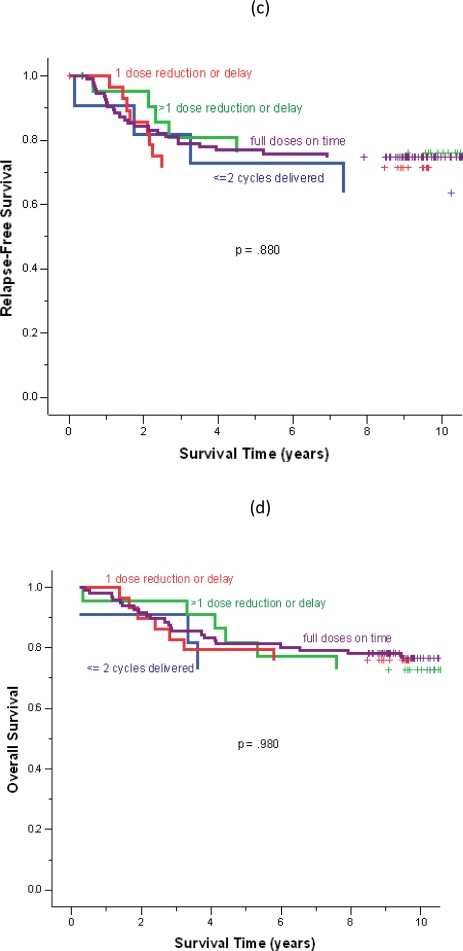

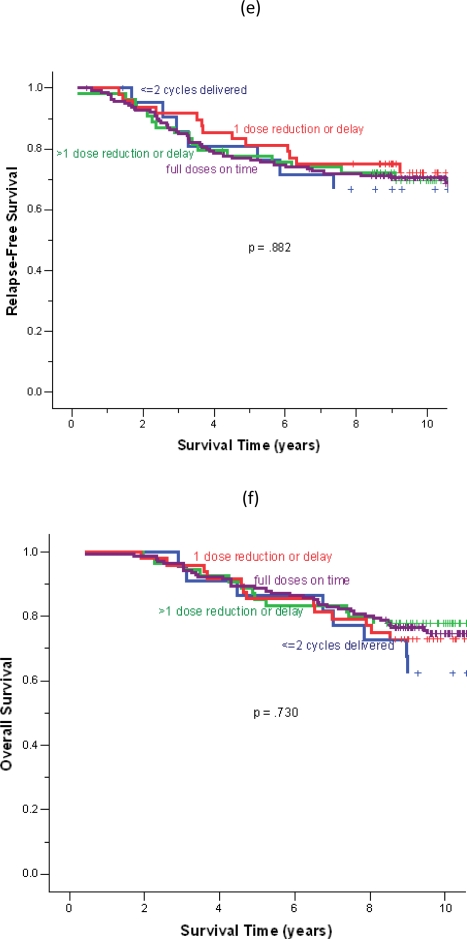
(a) relapse-free survival for all cases, (b) overall survival for all cases, (c) relapse-free survival for ER(–) cases, (d) overall survival for ER(–) cases, (e) relapse-free survival for ER(+) cases, (f) overall survival for ER(+) cases

The median follow-up duration of the total cohort of patients was 9.6 years. During adjuvant treatment, 45% of patients required at least one dose reduction or dose delay. The clinical end points of RFS, BCCS and OS were not significantly different among the four cohorts (Table 2). The eight-year survival estimates for cohorts 1–4, respectively, were: RFS 72%, 74%, 74%, 68%; BCSS 80%, 77%, 82%, 80%; and OS 78%, 76%, 80%, 77% (Figure 1a and b). Survival was further analysed based on endocrine responsiveness. For the 158 (33%) ER(–) breast cancer cases included in the study, the eight-year RFS, BCSS and OS were also not significantly different among the four cohorts (Table 2, Figure 1c and d). Likewise, the eight-year figures were not significantly different among the four cohorts for the 267 (55%) ER(+) breast cancer cases (Table 2, Figure 1e and f). Overall, 65.3% of the patients with ER(+) breast cancer were treated with adjuvant tamoxifen (Table 3). The differential prescription of tamoxifen across the cohorts, in particular cohort 2 versus cohort 1, was due to the provincial guidelines, which in that period, did not recommend adjuvant tamoxifen in pre-menopausal patients with node negative breast cancer (higher rate of node negative breast cancer in cohort 1 versus cohort 2 because the clinical benefit of adjuvant tamoxifen in pre-menopausal breast cancer had not yet been ascertained.

## Discussion

Modern adjuvant breast cancer treatment is increasingly dependent on biological features to guide prognosis and therapy. While the classic clinical prognostic factors such as lymph node status, grade and stage remain important, prognostic and predictive factors such as endocrine responsiveness (ER and PR status), as well as the HER-2/neu status of tumours are now routinely used to further refine therapy. Hormonal therapies, such as tamoxifen, aromatase inhibitors and ovarian ablation, and monoclonal antibody therapy using trastuzumab are therapeutic options, which have been shown to improve patient outcomes and are commonly used adjuncts to chemotherapy in properly selected patients [13]. While there is a breadth of chemotherapy options (e.g. anthracycline-based, anthracycline and taxane-based, dose dense) the AC regimen remains a commonly used form of chemotherapy. It may be considered as the sole treatment modality in low-risk, endocrine non-responsive disease, or more often, it will form part of a treatment plan such as AC for four cycles followed by a taxane, with or without subsequent hormone therapy or trastuzumab. Therefore, it is important to understand the possible impact of dose modifications of AC therapy on clinical outcomes.

Randomized controlled trials have shown that not only the cumulative dose of chemotherapeutic agents, but also the delivery schedule may be important for improving breast cancer outcomes [14, 15]. Early results of the CALGB 9741 trial suggest that increasing the dose density of AC for four cycles followed by paclitaxel for four cycles (AC+T) through the use of haematopoietic growth factors to deliver the regimen every two weeks, compared to the identical regimen every three weeks, improves RFS and OS in women with node positive breast cancer [15]. The absolute benefit on RFS and OS at three years was 4% and 2%, respectively, for the dose dense regimen, indicating a small, though significant improvement. Women with lower risk, node negative early breast cancer were not included in this study. Although they may potentially benefit from the addition of a taxane to the dose dense adjuvant anthracycline regimen (AC+T), the absolute gain is expected to be even smaller in this patient population [5, 15, 16]. For these lower-risk women, a regimen such as standard dose AC for four cycles is still quite appropriate. In fact, a recent trial (E2137) compared concurrent doxorubicin and docetaxel for four cycles to AC for four cycles in 2889 women with early breast cancer (of which 65% were node negative), and demonstrated no difference in DFS or OS between the two regimens [17].

Although generally well tolerated, AC can still lead to toxicity and dose reductions or delays. A survey of community oncologists across the United States has reported that of 6849 women with primary breast cancer treated with AC, 29% experienced a dose reduction of ≥15% or a delay of ≥7 days at some time during their course of treatment [12]. That study did not assess the impact of this on patient outcomes. In our study of patients treated with four cycles of adjuvant AC, dose modifications (of ≥25% in any cycle) or delays (of ≥5 days in any cycle) were experienced by 45% of patients.

Recognizing a ***threshold*** effect of both cumulative dose and dose intensity of anthracyclines, and in light of emerging evidence that dosing schedules may impact on outcome in women with node positive breast cancer, oncologists are concerned that any single-dose modifications or delays, even for the management of asymptomatic neutropenia, will compromise clinical benefit. Oncologists are known to utilize haematopoietic growth factors if dose reductions or delays in adjuvant anthracycline-based chemotherapy have been required [12]. Recent data also suggest that chemotherapy dose intensity may be particularly important for patients with ER(–) disease [18]. However, our retrospective population-based analysis of women with early stage breast cancer treated with up to four courses of standard adjuvant AC has not identified a relationship between DI and clinical outcome despite nearly a decade of follow-up. When endocrine responsiveness was considered no difference in outcome was appreciated between the four cohorts.

The definitions of dose reductions and delays in this study reflect the actual modifications commonly used by medical oncologists at the BCCA in managing treatment related toxicity (haematological and non-haematological). The four cohorts were designed to identify a relationship between incremental dose adjustments (and hence, intensity) and outcome. We specifically included a cohort of patients with only one dose adjustment during their treatment course, in order to assess the impact of a single-dose modification on clinical outcome. The lack of difference in outcome between patients in cohorts 1 (full dose without delays) and 2 (one dose adjustment) suggests that when a single reduction in dose intensity (whether through dose delay of ≥5 days or dose reduction of 25%) is required, it is reasonable to refrain from adding haematopoietic growth factors for subsequent cycles, if the only intention is to maintain dose intensity. This is further supported by the fact that there is again no statistical difference in outcome between cohorts 1 and 3 (>1 dose reduction or >1 dose delay, and at least three cycles of chemotherapy), suggesting that those patients who go on to have a second dose adjustment for ongoing toxicity still have similar outcomes. These results reflect reduced DI for only four cycles of standard dose AC, and may not necessarily hold true for newer generation 6–8 cycle chemotherapy regimens (such as TAC, CEF, CVAP-D, AC-Docetaxel) currently being utilized as adjuvant or neoadjuvant therapy in high-risk breast cancer [16, 19–22].

Our results may be explained by the small difference in relative DI (RDI) between the cohorts (cohort 1, RDI = 100%; cohort 2, RDI = 93.7–95.7%; cohort 3 (e.g. if two cycles of chemotherapy are delivered with a 25% dose reduction), RDI = 88.9%). Although the size of our study cohorts may limit the statistical power to detect a small difference in outcome, the long duration of follow-up, as well as the population-based nature of these data suggests that one single-dose reduction or dose delay during a planned four-cycle regimen of adjuvant AC chemotherapy can be undertaken with minimal detriment to the long-term outcomes and without need for haematopoietic support.

While cohort 4 appears to have received the lowest RDI, the true treatment delivered to these patients is not known. Some patients may have received treatment at other cancer treatment centres, or switched to alternative regimens. The small number of patients in cohort 4 (*n*=39) prohibits definitive conclusions from being drawn. However, the FASG 05 and CALBG 8541 trials clearly demonstrated a DI of ≤50% of the anthracycline lessens the benefit of adjuvant chemotherapy.

There is an important conceptual difference between the intentional, ***a priori*** reduction of the delivered dose (such as the study design of the FASC 05 and the CALGB 8541 trials [2, 3], and dose reduction specifically for treatment-related toxicity. Prospective trials that demonstrate a ***threshold*** effect of an anthracycline prove that on a population basis, a certain cumulative dose and/or dose intensity is required to improve outcomes. For doxorubicin, the threshold is generally believed to be around 60 mg/m^2^ when the drug is delivered on a three-weekly schedule, however, whether the true threshold dose lies somewhere just below 60 mg/m^2^ is not known. Importantly, the effective ***threshold*** doses for ***individuals*** within a population are not known. Presumably, some patients may have a threshold at a dose less than 60 mg/m^2^. It has been suggested that haematological toxicity may serve as a marker to identify the appropriate threshold DI for an individual patient [23]. This has not been evaluated in a prospective fashion; however, retrospective data examining the impact of treatment-related leucopoenia and outcome suggest that patients who experience haematological toxicity may in fact have better clinical outcomes [10, 11, 24]. Conversely, those who do not experience adequate haematological toxicity may be receiving less than their optimal individual threshold dose of chemotherapy. If this model is applied to the context of treatment-related toxicity, it would suggest that patients experiencing toxicity were initially receiving a dose at or in excess of their individual threshold. If the model is assumed to be accurate, dose reductions and delays for toxicity can be applied and are unlikely to impact on outcome.

An association between hormone responsiveness and DI has recently been reported [18, 25]. Patients with ER(–) breast cancer who receive <85% of their protocol specified dose of CMF chemotherapy for cycle 1 of treatment had worse DFS and OS compared to those patients who received >85% of their protocol-specified dose for cycle 1. While our study did not show a difference in outcome based on ER status, conceptually the question addressed in the study by Colleoni *et al* [18] and our study is different. In our study, dose intensity was reduced only for treatment-related toxicity, while the Colleoni *et al* analysis considered patients whose doses were reduced ***a priori***, for cycle 1 of treatment. The possibility that many of these patients could have tolerated higher doses and were in fact being treated below their individual chemotherapy threshold cannot be ruled out.

Several studies are frequently cited to illustrate the relationship between DI and clinical outcome. In these studies, patients who received <85% of their scheduled chemotherapy dose had worse clinical outcomes [8, 24]. It is important to note that these results are based on retrospective analyses of clinical trials conducted in the late 1970s and 1980s, using a non-anthracycline-based regimen of CMF. The dose adjustments for toxicity, especially haematological toxicity, applied to these studies can be considered conservative by today’s standards. For example, in the retrospective analysis of Bonadonna *et al* [8], chemotherapy doses were reduced by 50% in the presence of Grade 1 myelosuppression. Fifty per cent dose reductions are rarely used in modern day chemotherapy. By reducing the dose so markedly at the first occurrence of Grade 1 toxicity, the researchers may have reduced the dose intensity below the individual’s threshold. That is, if smaller increments could have been used, it is possible that more patients would have received a greater proportion of their scheduled dose, and the results of the analysis may have been substantially different.

Prospective randomized trials to define the impact of toxicity-related reductions in DI on clinical outcomes are unlikely to be conducted. Although reduced dose intensity of AC chemotherapy for early stage breast cancer is common, the results of this population-based study indicate that a single-dose reduction or delay of AC chemotherapy for women with early stage breast cancer may not impact on clinical outcomes, and require no additional interventions, particularly in lower risk patients such as stage I breast cancer. Furthermore, retrospective studies from either clinical trials or large population-based databases may help to further confirm or refute this hypothesis.

## Figures and Tables

**Table 1:**
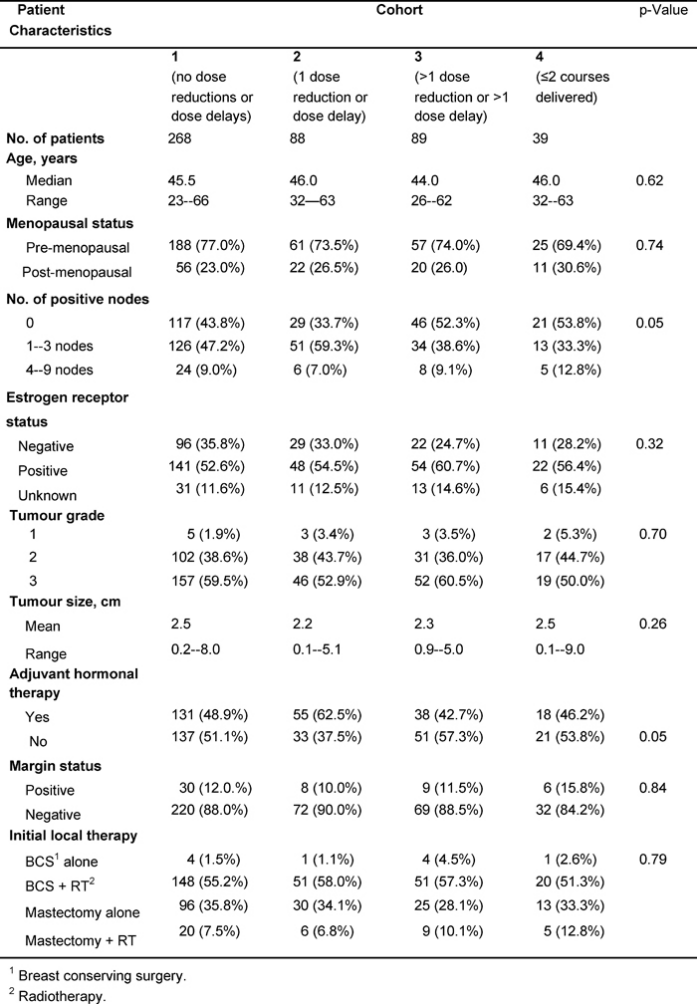
Patient characteristics at the time of diagnosis

**Table 2:**
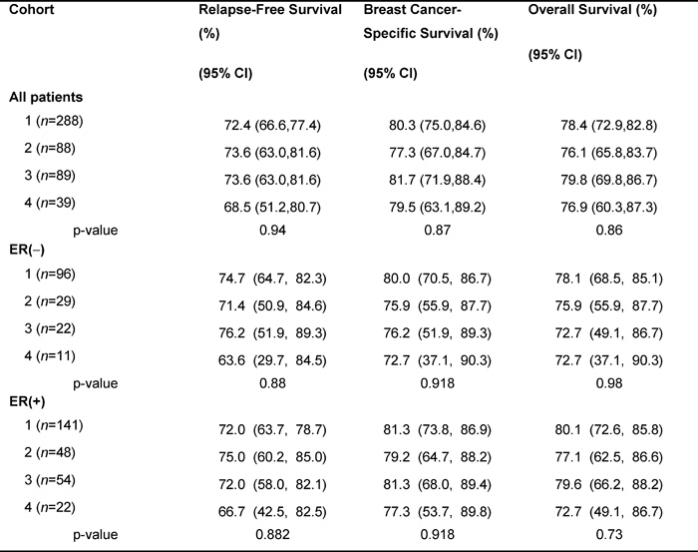
Eight-year survival data

**Table 3:**
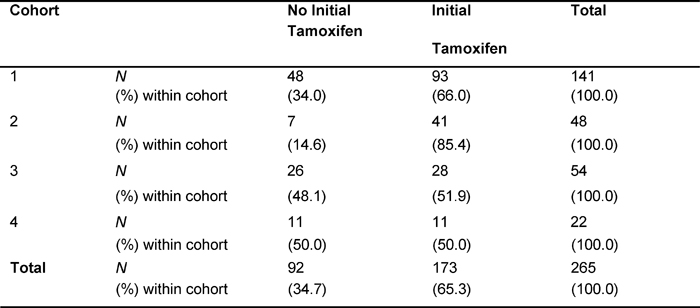
Proportion of patients with ER(+) breast cancer who received tamoxifen (*N*=265)
